# A novel methodology to characterize beat-to-beat alternations in whole-cell calcium currents

**DOI:** 10.1371/journal.pone.0339890

**Published:** 2026-01-28

**Authors:** Xavier Marimon, Miguel Cerrolaza, Carmen Tarifa, Leif Hove-Madsen

**Affiliations:** 1 Department of Strength of Materials and Structural Engineering, Universitat Politècnica de Catalunya, (UPC- BarcelonaTECH), Barcelona, Spain; 2 Institut de Recerca i Innovació en Salut (IRIS), Barcelona, Spain; 3 School of Engineering, Science and Technology, Universitat Internacional de Valencia (VIU), Valencia, Spain; 4 Spanish National Center for Cardiovascular Research (CNIC), Madrid, Spain; 5 Instituto de Investigaciones Biomédicas de Barcelona (IIBB-CSIC), Barcelona, Spain; 6 Biomedical Research Institute Sant Pau (IIB Sant Pau), Barcelona, Spain; 7 Centro de Investigación Biomédica en Red, Enfermedades Cardiovasculares (CIBERCV), Madrid, Spain; University of Minnesota, UNITED STATES OF AMERICA

## Abstract

An automated high-throughput procedure to quantify the degree of electrical beat-to-beat alternants in human atrial myocytes from electrophysiological recordings of the transmembrane ion currents of calcium ions (*Ca*^*2+*^) is presented and discussed. The patch clamp technique in whole-cell mode was used to record the myocyte calcium signal. A database consisting of 24 patch clamp signals (*N* = 24) of which 13 had a uniform behaviour and 11 had an alternating behaviour, was created. Several features were computed to characterize the transmembrane ion currents: peak amplitude, time constants, and area under the ion current trace. The presented algorithm includes a feature detector whose accuracy has been validated using simulated calcium signals generated by an electrical model that accurately represents the cardiomyocyte behaviour in a patch clamp experiment. Among these calculated features, a new index measure, called the “alternation index”, is proposed in this work to quantify the degree of electrical beat-to-beat alternants. The index has been shown to be a robust measure (*p* ≤ 0.01 **) for cell detection with an alternating pattern. Good agreement was observed with alternations in other calculated features like the measurement of the inactivation of L-type calcium current (*I*_*ca*_) or the tail current (*I*_*Tail*_) generated by calcium extrusion upon repolarization.

## 1. Introduction

Electrical alternans (EA) is clinically observed in electrocardiographic (ECG) recordings as beat-to-beat alternations in the amplitude, duration or direction of the relevant morphological attributes of the ECG signal such as the P wave, PR segment, QRS complex, RR interval, ST segment, and T wave [1,2]. In 1909 EA was first described by Hering [3]. About forty years later, Kalter and Schwartz [4] made the first description of EA with the ECG signal. For instance, ST-elevation alternans is known to be produced by abnormalities in the conduction of Purkinje’s fibers and is associated with pathologies such as ischemia, atrial fibrillation (AF), and acute myocardial infarction [5–8]. Similarly, T-wave alternans is associated with pathologies such as heart failure and severe ventricular arrhythmias [1,9,10]. Furthermore, alternating voltage in QRS amplitudes is often caused by pericardial effusion and associated with atrioventricular reentry tachycardia (AVRT) [11].

Atrial fibrillation is the most common cardiac arrhythmia in clinical practice [12] and the one that generates the most visits to emergency services. This arrhythmia occurs when individual cardiac muscle fibers contract asynchronously. It affects 1–2% of the general population, and its prevalence increases with age in both men and women [13,14]. Electrical alternans is indicative of heart disease [7,15] and atrial fibrillation [4,5,16] and although it is well studied at a complete organ level, much research remains to be done on the origin of these irregular conduction phenomena at the molecular to the intracellular, cellular, and tissue scales. It has been observed that, in the human atrium, electrical alternation could precede and induce atrial fibrillation, and at the cellular level atrial fibrillation has been associated with a decrease of L-type calcium current *I*_*Ca*_ [17,18] and an increase in the spontaneous calcium releases of the sarcoplasmatic reticulum (SR) as shown by Hove-Madsen et al. [19] and Kulkarni et al. [20] which may have its origin in genetic, molecular or subcellular alterations [21–23].

The study of calcium fluxes is of particular interest, as some authors point out, that electrical alternation depends on the calcium concentration *in vivo,* where it can be induced by lowering calcium [24], and prevented by calcium administration [10]. Calcium alternans have also been described at the cellular level in human atrial myocytes, where calcium content, *I*_*Ca*_, and the amount of calcium released by the SR have been shown to contribute to the development of calcium alternans [24–27]. Moreover, to address this issue, whole membrane currents have been measured with a patch-clamp technique, which allows for studying beat-to-beat alternation in ionic currents flowing across the sarcolemma. Therefore, most measures to characterize calcium current alternans focus on describing the morphology and kinetics of the current. Although most patch-clamp amplifiers have commercial software for the analysis of ionic currents, these, being a closed and proprietary system, have little or no degree of adaptation to a particular type of experimentation. Hence, this study developed and validated an algorithm that recognizes beat-to-beat alternations in whole membrane currents and characterizes the features that can be used to describe alternating pattern such as the peak amplitude, area and time constant.

The mechanisms of calcium handling have been extensively studied using calcium imaging techniques derived from confocal microscopy recordings, alongside computational optical mapping methods based on image processing. These approaches have been employed to detect alternans phenomena in perfused mouse hearts within a 2D plane [28–38]. Similar studies have been conducted in perfused guinea pig heart models [39–41]. However, investigations at cellular level are limited to studies focusing on fluorescence imaging in isolated myocytes [27,36,42,43].

As of today, unfortunately, there is no automatic alternants detection system from time series of calcium currents obtained through electrophysiological recordings at the cellular level. This has led to interest in developing a computational framework for characterizing alternans phenomena from calcium signal recordings, rather than from the widely studied calcium imaging techniques. By relying on ionic current signals, we can investigate conduction abnormalities at the ion channel level, whereas the optical techniques discussed earlier are limited to studying alternans at the tissue or whole-organ level.

## 2. Materials and methods

### 2.1. Atrial myocyte preparation

Human atrial cardiomyocytes (Human Atrial Myocyte, HAM) were isolated as previously described [19] (see Suppl. Material). Only elongated cardiomyocytes with clear cross striations and without granulation were used for experimentation. All samples were taken with informed consent of the donors. The experimental protocol has been approved by Hospital de la Santa Creu i Sant Pau Ethics Committee and complies with the Helsinki Declaration of Ethical Principles of the World Medical Association [44]. Patients who received treatment with calcium antagonists were excluded from the study.

### 2.2. Patch-clamp recording

The experimental setup and protocol used to record calcium currents across the cardiomyocyte membrane is described herein. A total of 24 experimental patch clamp signals were recorded (N = 24).

#### 2.2.1. Patch-clamp setup.

Calcium currents (*Ca*^*2+*^) were recorded using the perforated patch-clamp technique in freshly isolated myocytes in the whole-cell configuration. Pipette resistance varied from 1–2.5 mΩ. Cells were discarded if the serial resistance of the membrane *R*_*m*_ was greater than 6 times the pipette resistance *R*_*p*_*.* A Heka EPC 10 USB amplifier [45] was used for the recording, which has an analog-to-digital converter (ADC) with a maximum sampling frequency, *Fs*_max_, of 200 kHz. Series-resistance compensation was not performed.

#### 2.2.2. Electrical stimulation protocols and signal description.

**Fig 1:**
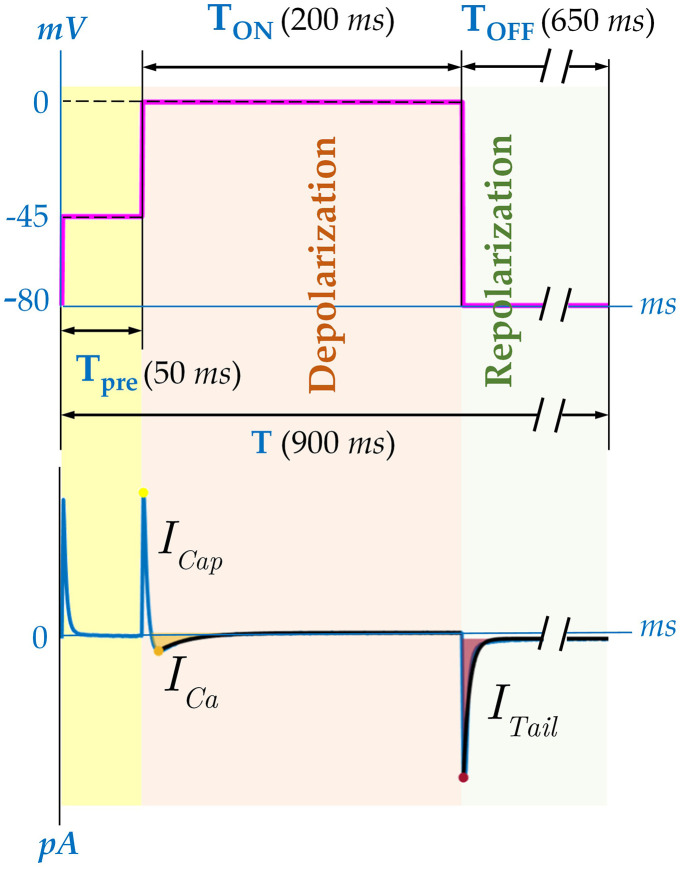
Suggested voltage-clamp stimulation protocol and corresponding ionic currents recorded in human atrial myocytes. A schematic representation of the voltage steps applied to elicit L-type calcium currents (*I*_*Ca*_) and tail currents (*I*_*Tail*_). A 50 ms prepulse from −80 to 45 mV is used to inactivate the sodium current (*I*_*Na*_). Subsequently 200 ms depolarization from −45 to 0 mV elicits first a fast outward capacitive current (*I*_*cap*_) followed by and inward calcium current (*I*_*Ca*_). Upon repolarization to –80 mV, a transient inward tail current (*I*_*Tail*_) are recorded. This protocol was repeated for 30 consecutive pulses to assess beat-to-beat variability in calcium current responses.

To address the impact of the stimulation frequency on whole membrane calcium currents, a stimulation protocol depicted in Fig 1 was used. To eliminate contamination from potassium currents, potassium was substituted by cesium in bath and pipette solutions (see Suppl. Material). A 50 ms pre-pulse from −80 mV to −45 mV was used to eliminate sodium currents (see Fig 1). Subsequently, the membrane potential was depolarized from −45 mV to 0 mV for 200 ms in order to elicit the L-type calcium current *I*_*Ca*_. Finally, the membrane potential was returned to −80 *mV* inducing an inward tail current, *I*_*Tail*_. This stimulation protocol was repeated 30 times (*Nsweep* = 30) at stimulation frequencies ranging between 0.5 and 2 Hz.

**Fig 2:**
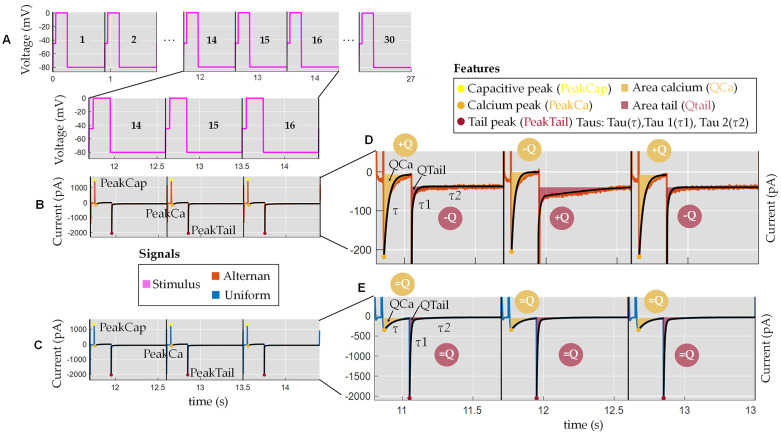
Features used for the alternating regime detection in patch-clamp calcium current recordings in the human atrial cardiomyocyte. **A.** Pulsed voltage-clamp protocol applied to the cardiomyocyte across 30 sweeps. **B–C.** Representative consecutive current traces showing uniform and alternating beat-to-beat responses. The algorithm automatically identifies key features in each sweep, including: capacitive peak (PeakCap),calcium peak (PeakCa), tail peak (PeakTail), area of calcium current (QCa), area of tail current (QTail) and time constants (taus):tau (τ), tau 1 (τ₁), tau 2 (τ₂). **D.** Detailed view of alternating response. **E.** Detailed view of uniform response.

To study the impact of pharmacological manipulations, the stimulation frequency, or other interventions expected to the beat-to-beat response, the algorithm was designed to determine different features of the whole membrane currents elicited by the stimulation pulses as displayed in Fig 2A. A total of 24 patch-clamped cells (*N* = 24) were studied, with 13 of them having a uniform response and 11 of them having an alternating response. For each response, the stimulation protocol with a variable pulse was repeated successively 30 times (*Nsweep* = 30). The charge carried by the tail current *Q*tail was measured as the area under the curve starting 2 ms after repolarization to −80 mV. A total of 6 features were computed for each sweep. These features are: *I*_*Ca*_ peak (*PeakCa*), *I*_*Ca*_ area (*Q*_*Ca*_*)*, *I*_*tail*_ area (*Q*_*Tail*_), tau *I*_*Ca*_ (*τ*), tau1 *I*_*tail*_ (*τ*_1_), *tau2 I*_*tail*_ (*τ*_2_). Therefore, a feature matrix of size 30·6 = 180 values is obtained for each cell signal. Representative ion currents are shown for cardiomyocytes with an alternating (Fig 2B) or a uniform response (Fig 2C).

A total of six features reflecting the activity of mechanisms that regulate the induction of alternating responses in human atrial myocytes were computed for each sweep (see [25] for details). Briefly, these features characterized the calcium current, *I*_*Ca*_, and are: *I*_*Ca*_ peak (*Peak*_*Ca*_) and *I*_*Ca*_ area (*Q*_*Ca*_) that initiates the calcium transient; tau *I*_*Ca*_ (*τ*) that is modulated by the calcium released from the SR and therefore will alternate if there are alternations in the released calcium; and finally the features that characterize the tail current, *I*_*tail*_, that are: the tail current *I*_*tail*_ area (*Q*_*Tail*_) that reflects the calcium extruded from the cell by the NCX and an indirect measure of the calcium transient, decay of the tail current tau *I*_*tail*_ (*τ*_1_) and tau2 *I*_*tail*_ (*τ*_2_) that depends on the activity of the NCX and calcium reuptake in the SR by SERCA, which in turn determines the restitution of the calcium transient on the subsequent beat and hence the triggering of alternation. The charge carried by *I*_*Ca*_ (*Q*_*Ca*_) and the tail current *Q*_*tail*_ were measured as the area under the curve starting 2 ms after depolarization to 0 mV and repolarization to −80 mV, respectively.

**Fig 3:**
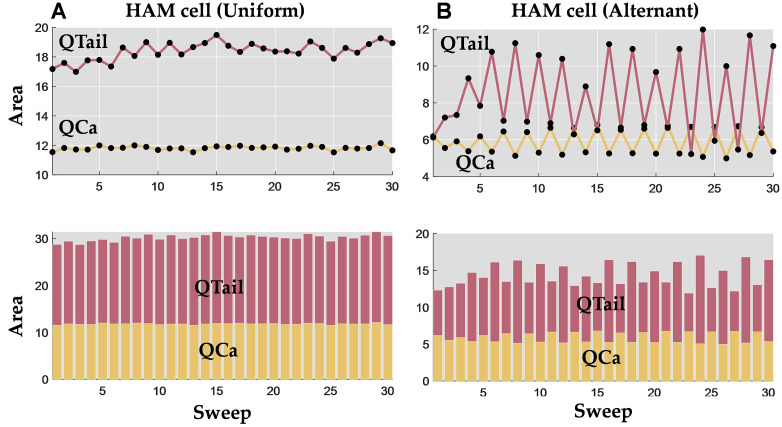
Variation in calcium current area (QCa) and tail current area (Qtail) features in human atrial in cardiomyocytes with uniform and alternant electrical phenotype response. **A.** Uniform response with stable current area (QCa) and tail current area (Qtail) amplitudes across 30 sweeps. **B.** Alternating response showing beat-to-beat alternations with current area (QCa) and tail current area (Qtail) out of phase. This relationship reflects the coupling between sarcolemmal calcium influx and sarcoplasmic reticulum (SR) calcium release.

Fig 3 shows the variation of the area parameters for a representative human atrial myocyte with a uniform and alternating response regime. Notice that the alternating response is characterized by clear alternations in the *Q*_*Ca*_ and *Q*_*tail*_, with amplitudes that are out of phase, i.e., a big *Q*_*Ca*_ amplitude is followed by a small *Q*_*tail*_ and vice-versa (see Fig 3B). This occurs because *Q*_*tail*_ reflects the amplitude of the calcium released from the SR. Calcium released by the SR, in turn, feeds back negatively on *I*_*Ca,*_ resulting in an inverse relationship between *I*_*Ca*_ and *Q*_*tail*_. In the context of signal processing, it is essential to address uncertainties and inaccuracies. Recent advances in fuzzy logic, in particular the fuzzification of data by similarity, offer a promising approach to address these challenges. This method allows similar data points to be merged into groupings from which fuzzy features can be extracted, as demonstrated by Versaci et al. [46] for the characterization of anisotropic materials in civil engineering.

Although our study does not implement fuzzy logic tools, these methods have proven to be effective in other fields for real-time applications with low computational load [47]. The transversality of fuzzy logic suggests that such techniques could be directly applicable to our work, potentially increasing the robustness and accuracy of the measurement indices in future developments.

### 2.3. Modelling the patch clamp experiment

To validate the features extracted for characterizing the beat-to-beat behavior of cardio-myocytes, such as determining whether they exhibit a uniform or alternating response, a detailed numerical model is required. This model should be able to describe the biophysics of the cardiomyocyte and the electronic system used to acquire the current signal from the cell membrane in a realistic way.

#### 2.3.1. Electrical model description.

**Fig 4:**
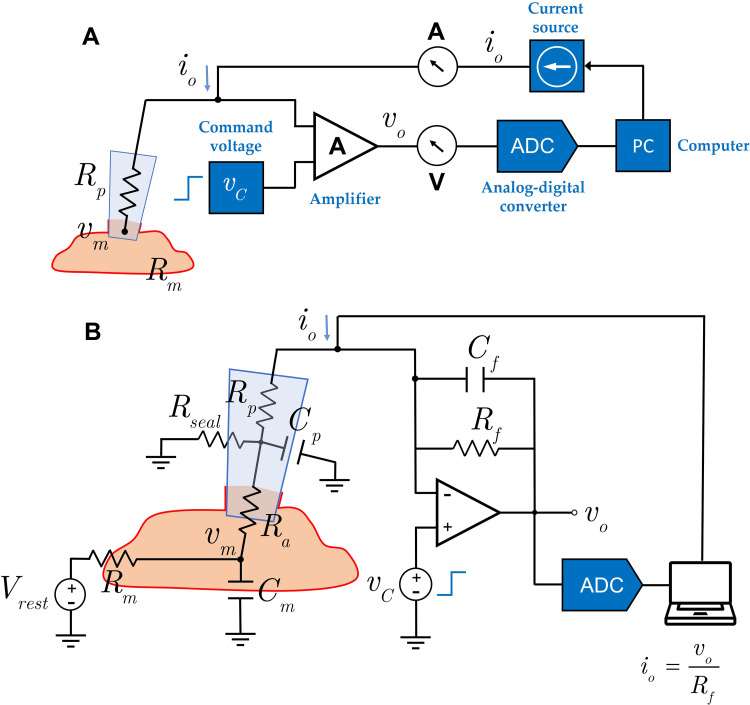
Electrical model of the whole-cell patch-clamp setup. A. Block diagram of a whole-cell voltage-clamp patch-clamp configuration using a single pipette. Electrical model of the whole-cell patch-clamp configuration used for parameter validation. B. The model combines a biophysical representation of the cardiomyocyte membrane and the recording hardware, including pipette resistance, amplifier feedback, and filtering circuits. This hybrid model generates realistic synthetic currents used to validate the feature extraction accuracy of the algorithm.

To validate the parameters accuracy obtained in our computational framework, a zero-order model (electrical model) was created to generate realistic synthetic signals that describe the pulsed clamp protocol described in Section 2.2.2. The model presented to describe the patch clamp experiment in whole-cell configuration is composed of two main parts as show in Fig 4. The first part models the biophysics of the cardiomyocyte, which involves the electrical processes and artifacts that occur within the cell membrane. The second part of the model was dedicated to modelling the electronic system used to acquire the current signal from the cell membrane. This part of the model takes into account the amplifies and filters which are used in the hardware of the patch-clamp device. For the modelling of the excitable cell, a classical electrical model has been used and adapted from several previous works [48–54] with the nominal values of the cardiomyocytes obtained through their experimental measurement or through bibliography [55,56]. The hardware of the patch-clamp amplifier has been modelled based on the technical specifications of the Heka amplifier used in this study.

The Heka amplifier has two configurable filters: the filter 1 is a band-pass filter (BP) with Bessel typology, while filter 2 is a low-pass filter (LP) with selectable Bessel or Butterworth typologies. Filter 1 can be used in series with Filter 2 or as separate filters. In our experiment, only the filter 2 has been selected, as a low-pass filter of order 4 (*n* = 4) with Bessel typology. The filter is used to reduce the oscillations of the amplifier output. The Heka amplifier has three feedback resistors *Rf that* can be selected: Low gain range (5 MΩ), Medium gain range (500 MΩ) and High gain range (50 GΩ). In our case the option ‘Medium’ gain range was selected, which is mainly used for whole-cell recordings.

The electrical model parameters have been adjusted to match the experimentally measured parameters. The electrical circuit has been implemented and simulated using the LTspice software v17.0 [57]. The biophysical electrical circuit used to simulate the patch clamp experiment is shown in Fig S1 in S1 File (see the Suppl. Material). The nominal values of the electronic components have been obtained from the manufacturer’s datasheet. A detailed explanation of each parameter can be found in the Suppl. Material of the article. The electrical variables used in the in-silico model of Fig 4 are summarised in Table 1 below:

**Table 1:** Electrical parameters used in the patch-clamp simulation using a zero-order model (electrical model). Simulation parameters have been obtained empirically from a real cardiomyocyte or through bibliography.

Part	Component	Nominal value
Cardiomyocyte	Membrane capacitance (*C*_*m*_)	55 pF
	Membrane resistance (*R*_*m*_)	500 MΩ
	Membrane voltage (*v*_*m*_)	−90 MV (rest)
Amplifier	Access resistance (*R*_*a*_)	15 MΩ
	Seal resistance (*R*_*S*_)	1GΩ
	Feedback capacitance (*C*_*f*_)	0 *F*
	Control voltage (*v*_*C*_)	0 a −80 mV
Pipette	Pipette resistance (*R*_*p*_)	0Ω
	Feedback resistance (*R*_*f*_)	500 MΩ
	Pipette capacitance (*C*_*p*_)	≈ 0 F
Filter (low-pass, *n* = 4)	Resistance 3 (*R*3)	1 MΩ
	Resistance 1 (*R*1)	50Ω
	Inductance 1 (*L*1)	2 mH
	Inductance 2 (*L*2)	4.3 mH
	Capacitance 1 (*C*1)	1.3 μF
	Capacitance (*C*2)	270 nF
	Capacitance (*C*3)	3.6 μF

#### 2.3.2. Electrical model validation.

Validation through simulations has been focused on depolarization, where the cell membrane potential becomes more positive, and it is an important step in the process of muscle contraction. Since only the synthetic model is used to validate the correct detection of the features in the current signal elicited by the stimulation pulse, it is not necessary to have a model that captures the alternation process, because we are looking for the features for each current response individually. Therefore, the model must be realistic in modelling the current response. In order to make the model more realistic, Gaussian White Noise (GWN) has been additively added to the simulated signals. The noise variance in the experimental signals has been inferred using the robust Median Absolute Deviation (MAD) noise estimator [29,58]. Then, a mean noise variance value of *σ*_noise_ = 188 has been obtained in the experimental signals. The estimated noise variance in the experimental signal is added to the synthetic signal following an additive GWN model: *x*_noise_ = *x* + *N* (0, *σ*_noise_) [59,60]. Here, the noise is modelled by a Gaussian distribution *N~*(0, *σ*_noise_) with zero mean and a standard deviation *σ*_noise_.

The accuracy of the model was assessed by computing the Mean Absolute Error (MAE) [61–63] between the numerically simulated current signal and the experimental signal. Furthermore, the normalized cross-correlation (xCorr) was computed as an additional index for assessing the quality of the model [64]. For each non-pathological experimental signal (uniform signal, *N* = 13), a simulated signal was obtained and compared with an experimental signal using the MAE and Xcorr index, giving a value of MAE = 1.8372 and Xcorr = 0.8925. These indices show a good agreement between the synthetic model and the experimental data, which means that the in-silico model is able to mimic the non-pathological signal response.

### 2.4. Computational framework description

**Fig 5:**
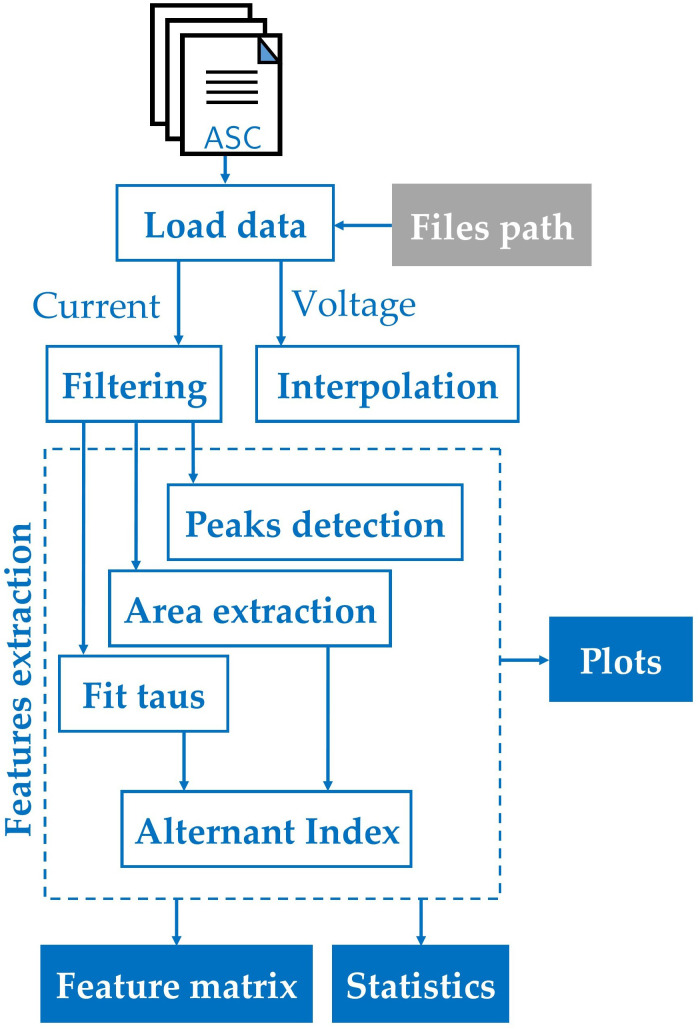
Flowchart of the computational framework for characterizing experimental patch-clamp signals under pulsed clamp voltage. The diagram starts by loading the data from the path definition, where the patch clamp event log files are stored. Then the current and voltage time series are extracted and properly pre-processed (filtering and interpolation). Then the current signal features are extracted. Finally, the data can be visualised, stored, or a basic statistic of the computed features can be computed.

Custom code was developed using MATLAB [65] to create an in-house computational framework for feature extraction from patch clamp recordings. The framework uses specific libraries such as the Signal Processing Toolbox and the Statistics and Machine Learning Toolbox. The full procedure flowchart is shown in Fig 5.

#### 2.4.1. Load data.

The patch-clamp system acquires the experimental data and saves it in an ASCII 7-bit encoded file (*.asc file). The program loads *.asc files from the indicated folder into the main program, decoding the information contained in each file to obtain the stimulation voltage and membrane current.

#### 2.4.2. Filtering.

The current is then filtered with a moving average filter to obtain a smoothed signal [66,67]. The filtered signal *i*_*filt*_(*n*) is obtained by convoluting the original signal *i*(*n*) with a rectangular window [68,69]


$$i_{filt}(n)=i\;(n)*W\;(n,L)$$ (1)

where *W*(*n*, *L*) is a rectangular window of length *L.* The length of the window is calculated by a heuristic method to attenuate 100*·k* percent of the energy of the original input current. In the present case, a value of *k =*0.02 has been used, which is equivalent to a window of length *L* = 2 samples.

#### 2.4.3. Interpolation.

The recorded stimulation signal differs from the actual stimulation signal due to small-potential change oscillations produced by electrical noise. Therefore, the recorded values of the actual stimulation voltage are interpolated with the theoretical values of the stimulation voltage (0 mV, −45 mV, and −80 mV), to obtain a signal as similar as possible to the reference signal. The interpolation is performed using the nearest-neighbor extrapolation method [70,71] where the extrapolated value to a point is the closest value to the values of an objective table by look-up table method (LUT), *V*_*targed*_, of 0 mV, −45 mV, and −80 mV.

#### 2.4.4. Feature extraction computation.

The electrophysiological features designed to characterize currents across the cardiomyocyte membrane are described herein. Together, these features provide a detailed picture of the transmembrane ion currents in human atrial myocytes, and allow for the identification of cells with an alternating pattern.

Today, a wide range of software for analyzing patch clamp recording data is available. While most of these tools are proprietary black-box solutions tied to specific electrophysiology equipment, such as Clampfit (Molecular Devices), Patchmaster [45] and Signal (Cambridge Electronic Design Limited), there are also free and open-source alternatives such as Eventer, WinWCP and Stimfit. Of these, Stimfit is the most widely used due to its embedded Python shell and has been extensively validated against gold standard electrophysiology software [72]. We compared our custom feature extraction system with Stimfit using our synthetic dataset of uniform response calcium signals (*N* = 13). The comparison showed an “unsuccessful fit” rate of 0% and a sum of squared errors (SSE) of 5.2x10-4, demonstrating the accuracy of our system. The average execution time for extracting calcium current features (such as amplitudes, areas, and time constants) with our custom script was 532 ± 4 (ms), tested on an Alienware X15 (Dell, Austin, Texas, United States) equipped with an Intel Core i9 2.50 GHz processor, 32 GB RAM, and an NVIDIA Titan X 12GB GPU.

***Calcium influx: Peak current features*:** The measurement of peak amplitude features involved the identification of the maximum peak value of the ion current trace. The peak detector measures the amplitude of the L-type calcium current *I*_*Ca*_ and the amplitude of the tail current of the Na^+^/Ca^2+^ exchanger (NCX), *I*_*Tail*_. The amplitude of the capacitive current *I*_*Cap*_ is of little interest at the diagnostic level.

However, this amplitude must be calculated to obtain the cardiomyocyte capacitance. Currents during the pre-pulse are discarded from the analysis. Peaks of the capacitive current *I*_*Cap*_ due to the capacitive behaviour of the cardiomyocyte cell membrane, although not of interest, are calculated to serve as a reference when calculating areas. Moreover, the capacitive current can be used as a reference point to avoid contamination by the capacitive current. The peak detector finds the local maxima by searching for points that are larger than the two adjacent points, while a series of peak amplitude and peak distance filters are applied to rule out too small peaks and ensure that the peaks have the right separation distance. The minimum peak prominence is defined as 10 pA, the minimum peak height is defined as 100 pA, the minimum peak separation between detected peaks is defined as 63*·T*_*s*_ s, and the minimum peak width is defined as 1.163*·T*_*s*_ s, where *Ts* is the sampling period. To detect negative peaks, i.e., local minima, the same technique to find local maxima is used but reversing the signal: *i*(*n*)*=i*(*n*)*·*(−1)*.*

***Ion channels inactivation: Time constant feature*:** Time constant features measured the rate at which the ion currents change over time. The parameter measured to characterize the inactivation rate is the time constant of the rise curve. This parameter was obtained by fitting the L-type calcium current curve to a simple exponential model. The case of tail current *I*_*Tail*_ is a bit more complex. Two types of adjustments have been evaluated. One based on a single exponential model and another model based on a double exponential (further details can be found in the Suppl. Material).

Since the coefficient of determination (or r-square) *R*^*2*^ value is not an appropriate measure of the quality and complexity of the model, the assessment of the Goodness-Of-Fit (GOF) of the two models was done by using the Akaike Information Criterion (AIC) [73–76]. AIC penalises both under-fitting and over-fitting, and the model with the smallest AIC is considered to be the optimum model [77]. The number of estimated parameters is *k* = 4 for the single exponential and *k* = 5 for the double exponential model. Using a dataset of 24 patch-clamped cells (*N* = 24) where the stimulus was repeated successively 30 times (*Nsweep* = 30), then we have *N*_*Tail*_ = 720 tail current signals to check the quality of our models (single exponential model or double single exponential model).

Table 2 shows the mean value of the AIC criterion in the tail signal for the case of the single exponential and the double exponential fit. As the value of the AIC criterion is low in the case of the double exponential model, this model was used to extract the features related to the tail signal *I*_*Tail*_. This way to proceed was consistent, since the inactivation curve obtained in the experimental tail data has two clearly differentiated dynamics: a fast dynamic *τ*_1_ and a slow dynamic *τ*_2_ (see Fig S3.B in S1 File).

**Table 2:** AIC values for the single and double exponential models.

Model	Mean AIC *N*_*Tail*_ = 720
Single exp. model	6.33e + 03
Double exp. model	5.07e + 03

***Calcium transport: Area features*:** The calcium transport across the cell membrane is calculated from the area under the curve of the calcium current *Q*_*Ca*_ during depolarization and from the area under the curve of the tail current *Q*_*Tail*_ during repolarization. To calculate *Q*_*Ca*_, the area under the capacitive peak curve must be eliminated by computing its intersection with the *y*-axis. *Q*_*Tail*_ is then obtained by numerical integration by using the trapezoidal method, which approximates the integral by dividing the area into small trapezoids. The temporal position of the detected peaks serves as a guide for calculating the area. For instance, the area of the calcium current is computed within a region of interest (ROI) that ranges from the position of the capacitive peak to the position of the *k* + 1 *nth* stimulation sweep. The mean amplitude value of the last *m* points of the ROI is used to find the currents steady state value. For the calcium current a value of *m* = 20 was used, while for the tail current *m* = 10. The steady state value defines the baseline and is a key parameter to correctly compute the area.

***Beat-to-beat alternans index*:** An alternation index was created in order to quantify the degree of beat-to-beat alternants based on the calculated features. The index is based on the variable *D*, which is the difference between consecutive values and is expressed as:


D(t)=m(t)−m(t−1) (2)

The alternation index CIdx ranges between 0 and 1, and was defined as the ratio of the mean value of the quadratic amplitude difference between two consecutive values *D* divided by the largest value of the quadratic amplitude difference between two consecutive values:


$$C\;Idx=\frac{\text{1}}{\left(N-1)\cdot \max(D{\left(t\right)}^{2}\right)}\cdot \sum_{t=1}^{N-1}D{\left(t\right)}^{2}=\frac{mean(D{\left(t\right)}^{2})}{\max(D{\left(t\right)}^{2})}$$ (3)

**Fig 6:**
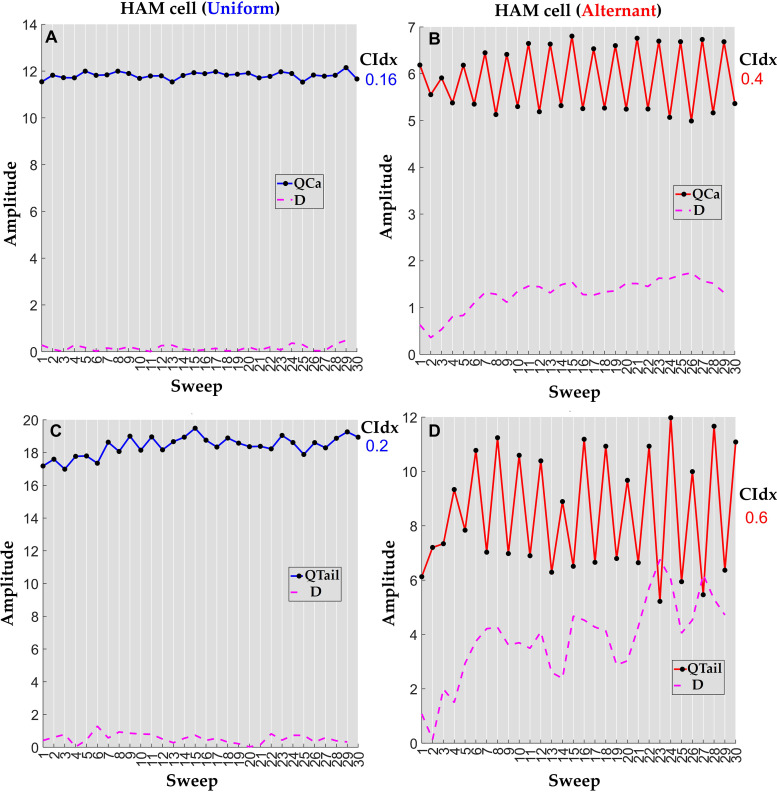
Calculation of the alternation index in representative uniform and alternant cell regimes subjected to a stimulation protocol of 30 consecutive voltage pulses. The variable D represents the difference between two consecutive values. **A, B.** Calculation of alternation index (CIdx) in a uniform and alternant cardiomyocyte response using calcium current area (*Q*_*Ca*_). **C, D.** Calculation of alternation index in a uniform and alternant cardiomyocyte response using tail current area (*Q*_*Tail*_).

Fig 6 shows an example of the calculation of the alternation index for a cardiomyocyte with uniform and alternating behaviour. The variable D can be any measured value presented in the previous sections, namely: peaks in L-type calcium current (*PeakCa*), peaks in the tail current (*PeakTail*), time constant (τ), slow time constant (τ_1_), fast time constant (τ_2_), calcium current area (*Q*_*Ca*_), and tail current area (*Q*_*Tail*_).

The Fig 6 shows the calculation of the alternation index using the AUC in the calcium current (*Q*_*Ca*_) and AUC in the tail current (*Q*_*Tail*_), as these are the measures that were found to discriminate most effectively between alternating and uniform regimes (see Section 3 Results and discussion).

The alternation index has been specifically designed to fulfil the critical requirements of a robust measurement index. First, it demonstrates high sensitivity to beat-to-beat variations by capturing even subtle differences in electrical activity through quadratic amplitude differences between consecutive values. Second, the index is specific to beat-to-beat alternation, ensuring that it effectively quantifies changes in cardiomyocyte behaviour without being influenced by unrelated signal fluctuations. Furthermore, the alternation index is normalized to a range of 0–1, which provides clear and consistent interpretation across various datasets and facilitates comparability under different experimental conditions. Also, it exhibits robustness by being applicable to multiple measured variables, including calcium currents: amplitudes, area, and time constants, thereby maintaining its precision across diverse contexts. Finally, the index is computationally efficient, and allows real-time analysis of large datasets. Together, these features confirm that the alternation index is a reliable and effective tool for quantifying beat-to-beat electrical alternations in cardiomyocytes.

#### 2.4.5. Feature extraction validation.

To assess if the extracted features are valid, an in-silico model was used. The features were extracted in two ways, automatic and semi-manual, and then tested during a depolarization stimulus pulse. The value of the features calculated semi-manually has been used as a reference value (ground truth) and is compared with the value calculated with the automatic feature extraction system (See Section 2.4: Computational framework description). Table 3 shows the relative error between the ground truth and the value calculated automatically.

**Table 3:** Errors in automatically calculated features in a synthetic pulse.

Features (depolarization pulse)	Error *ε* (%)
Area	0.86%
Peak amplitude	0.32%
Time-constant (Single exp. model)	1.27%

#### 2.4.6. Software outputs.

The software has several complementary modules for generating outputs that help the user to understand and visualize the results: a) a module to calculate the comparative statistics of all parameters between uniform and alternating conditions; b) a graphics module generates all the graphics in the software and store them as high-quality images in *.png format. This graphical output of the software includes the representation of calcium currents and stimulation voltage, as well as statistical results and c) a data export module where all input signals and calculated features are stored in a *.csv format file.

### 2.5. Data analysis and statistics

The results presented below have been subjected to statistical validation. The dataset created consists of 24 patch clamp signals (*N* = 24) of which 13 have a uniform behaviour and 11 have an alternating behaviour. As each cell has been subjected to 30 stimulation pulses, a total of 390 values are obtained for each feature with uniform behaviour and 330 values for each feature with alternating behaviour. The values used for the statistical analysis are expressed with the mean and the standard error of the mean (Standard Error of the Mean, SEM) as mean ± SEM. The notation used for statistical significance is as follows: *p*> 0.05 (ns), *p*≤ 0.05 (*), *p*≤ 0.01 (**), *p*≤ 0.001(***), and *p*≤ 0.0001(****). The *p*-values that meet *p*≤ 0.05 (*) are considered statistically significant. The data sample doesn’t follow a Gaussian distribution and, therefore, the non-parametric Wilcoxon rank-sum test was used. Experimenters were blinded to clinical information.

## 3. Results and discussion

This section presents the classical features that describe the dynamics of membrane currents (amplitudes, areas, time constants) in cardiomyocytes, also explaining whether they are useful to distinguish between a uniform and an alternating regime when a statistically significant difference between the two responses is found. However, although these features could be used as input to a classifier to discriminate between the two responses, they do not allow for measuring the degree of cardiomyocyte alternation. Hence, a new measure for quantifying the degree of alternation in a cardiomyocyte has been introduced, called the ***alternation index***.

In the following section, any discussion of physiological mechanisms or interpretations is presented strictly in a contextual manner and serves only to validate the algorithm’s performance. Our intention is not to provide an exhaustive physiological analysis of the observed phenomena, but rather to demonstrate that the algorithm’s outputs are consistent with established findings in the literature.

### 3.1. Peak amplitude features

**Fig 7:**
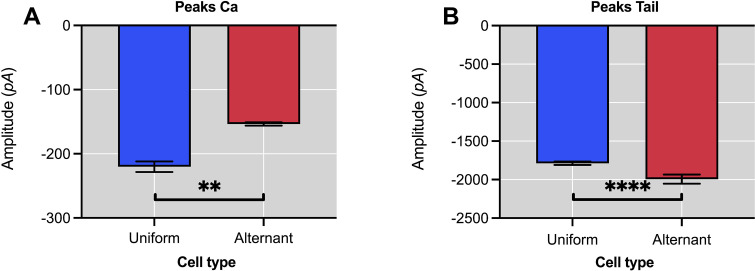
Comparison of peak amplitudes between uniform and alternant cardiomyocytes. **A.** Amplitude of the L-type calcium current (*I*_*Ca*_), is significantly lower (*p* ≤ 0.01) in alternant cells. **B.** Amplitude of the tail current (*I*_*Tail*_) shows corresponding variability. These differences suggest impaired calcium influx and altered SR calcium handling in alternant cells.

Statistically significant differences are observed in the amplitude measurements of the L-type calcium current, *I*_*Ca*_, between the cardiomyocytes with uniform and alternating responses, with a *p*-value of *p* ≤ 0.01 (**). The Fig 7A shows a decrease in the mean amplitude of the L-type calcium current peaks *I*_*Ca*_ in the cardiomyocytes that exhibit an alternating behaviour. This fact would be in agreement with the notion that alternation could precede the induction of atrial fibrillation, considering that it has been associated with a decrease in the L-type calcium current *I*_*Ca*_^17^ caused by an increase in the spontaneous and fluctuating release of calcium in the sarcoplasmic reticulum (SR) [19,78–80]. Therefore, it is shown how the alternation phenomena are regulated by a decrease in the calcium current *I*_*Ca*_ through the cardiomyocyte membrane [25].

### 3.2. Time constant features

**Fig 8:**
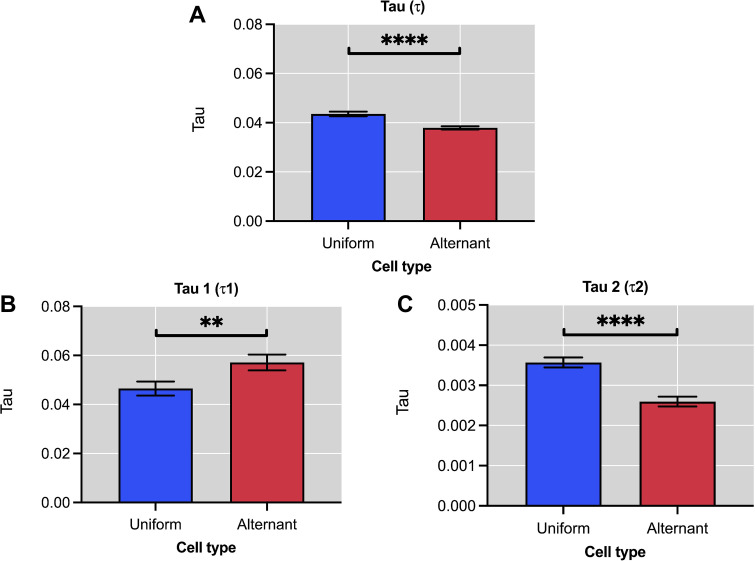
Time constant of ionic currents in uniform and alternating type cardiomyocytes. **A.** Time constant of calcium current (τ). **B.** Time constant of the fast tail current (τ_1_). **C.** Time cost of the slow tail current (τ_2_). Alternant cells exhibit prolonged τ₁ (*p* ≤ 0.01) and shortened τ₂ (*p* ≤ 0.0001), indicating altered kinetics of calcium extrusion and SR reuptake.

This section presented the time constant features obtained with the computational framework for the analysis of cardiomyocyte ionic currents. These measurements were useful for determining the inactivation dynamics of ion channels. Fig 8 shows the time constants calculated for cardiomyocytes with uniform response and alternating response.

It should be remarked that the time constant of the fast tail current *τ*_1_ in the uniform type signals was significantly smaller as compared to the alternating signals, *p* ≤ 0.01 (**) (see Fig 8B). Therefore, the dynamics in the first phase of inactivation (*τ*_1_) are, on average, faster in uniform type signals than in alternating signals. This increase in *τ*_1_ has also been observed in myocytes of patients with atrial fibrillation [17,81,82]. On the other hand, the time constant of the slow tail current, *τ*_2_, in uniform-type signals is significantly greater than in alternating signals, *p* ≤ 0.0001(****) (see Fig 8C).

### 3.3. Area features

**Fig 9:**
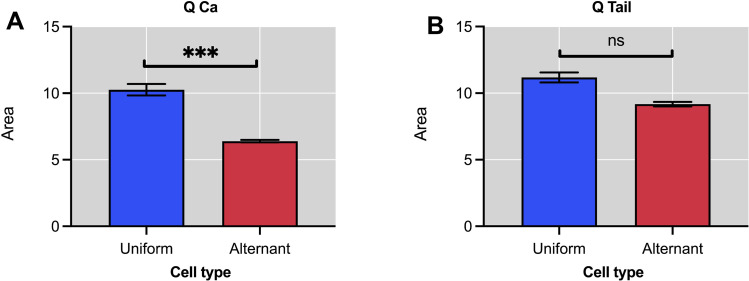
Areas under the current curves (AUC) for calcium and tail currents in uniform and alternant cardiomyocyte cells. **A.** Area under the calcium current curve (*QCa*). This area is significantly smaller in alternant cardiomyocytes (*p* ≤ 0.001), reflecting reduced calcium influx. **B.** Area under the tail current curve (*QTail*). Shows no significant difference. Together, these data highlight the asymmetrical regulation of calcium entry and extrusion in alternant responses.

This section discusses the area features obtained with the computational framework from the analysis of cardiomyocyte ionic currents in a patch clamp experiment. The measurement of the area under the curve (AUC) current is useful for studying calcium transport. Fig 9 showed measurements of area of L-type calcium current *Q*_*Ca,*_ which was related to the transport of calcium through L-type channels [83] and tail current *Q*_*Tail*_, which reflects the extrusion of the released calcium by the NCX exchanger [25,84,85].

Statistically significant differences were observed in the average values of areas between cardiomyocytes with uniform and alternating response, *p* ≤ 0.001(***), as displayed in Fig 9A. In contrast, no significant differences (n.s) were observed in average areas under the curve (AUC) of the tail currents of the *Na*^+^/*Ca*^2+^ (NCX) exchanger, *I*_*Tail*_, between cardiomyocytes with uniform and alternating response (See Fig 9B).

### 3.4. Alternation index

Finally, in this section, the results obtained with the new feature, called alternation index CIdx, are presented. This new index, in contrast to the classical features presented in the previous subsections, makes it possible to directly quantify the degree of beat-to-beat alternating behaviour of the cardiac cell. Since the index proposed in this study is a novel measure and no control references are available, we decided to apply the calculation of the index to the parameters for which indices are available that could have an alternating behaviour between the different sweeps of successive stimulations that are applied to the patch-clamp experiment.

The dynamics of the L-type calcium current *I*_*Ca*_ and sarcoplasmic reticulum calcium release reflected by tail current *I*_*Tail*_ play a relevant role in the regulation of the alternation phenomena. The L-type calcium current plays an important role in cardiac excitation-contraction coupling by allowing the influx of calcium ions into the cell, which triggers the release of calcium ions from the SR and initiates contraction. Abnormalities were observed in both L-type calcium current and SR calcium release and can lead to changes in the timing and magnitude of calcium release, which can result in beat-to-beat alternation [86,87].

**Fig 10:**
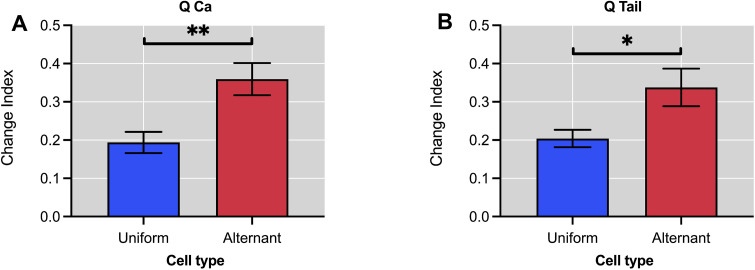
Alternation index, CIdx, for uniform and alternating type cardiomyocytes derived from calcium current area (*QCa*) and the tail current area (*QTail*). **A.** Alternation index calculated with the area under the calcium current curve (*QCa*). Differentiates alternating from uniform cells (*p* ≤ 0.01). **B.** Alternation index calculated with the area under the tail current curve (*QTail*), also shows significant discrimination (*p* ≤ 0.05).

Precisely, statistically significant differences were observed in the index calculated with the measurements of the areas under the curve (AUC) of the L-type calcium currents *I*_*Ca*_ between cardiomyocytes with uniform and alternating response with a p-value of *p* ≤ 0.01 (**), as shown in Fig 10A. Moreover, if the tail current *I*_*Tail*_ is used to calculate the alternation index CIdx, significant differences are generated with a p-value of *p* ≤ 0.05 (*), see Fig 10B.

## 4. Conclusions

A computational platform has been developed for the end-to-end characterization of experimental recordings of whole membrane currents in cardiomyocytes. The computational method allows the processing, visualization, and quantitative characterization of ionic currents that flow across the cardiomyocyte membrane.

The method proposed herein includes several steps, such as: a) graphic representation of ionic currents, thus allowing the visual inspection of the data, b) measurement of the inactivation of ion channels, which refers to the measurement of the process by which ion channels close or reduce their activity after being activated, c) measurement of calcium transport, which refers to the measurement of the movement of calcium ions across the cell membrane, d) measurement of classical kinetics features (peaks, areas, time constants) and e) measurement of beat-to-beat alternation using a new highly efficient index for detecting beat-to-beat alternating behavior in cardiomyocytes, called the “alternation index”.

Although the present algorithm was developed for analyses of calcium currents in isolated cardiomyocytes, it could be adapted to detect alternation in the shape of the action potential (rate of rise, overshoot, duration, decay). To achieve this, detection of alterations in specific features of the action potential signal should be incorporated. Therefore, to detect alternation in the action potential features related to sodium currents, *I*_*Na*_, features such as the rate of rise and overshoot should be incorporated into the algorithm. Likewise, the incorporation of features related to the activity of potassium, currents, *I*_*K*_, would allow detecting alterations in the overshoot, duration, or decay of the action potential.

In contrast to the classical kinetic features obtained from transmembrane currents, such as maximum amplitude, area under the curve, and time constants, which do not allow prediction of the degree of alternation in the cardiomyocyte, the new biomarker “alternation index” provides an effective one-shot measure to quantify the degree of alternation of the cardiomyocyte in beat-to-beat regulation. This new index proved particularly effective when used for area measurements, particularly in the area under the calcium current curve, *Q*_*Ca*_, and the area under the tail current curve, *Q*_*Tail*_.

## Supporting information

S1 File Experimental protocol and numerical modelling description. (PDF)

S1 Video Algorithm performance (uniform response). (MP4)

S2 Video Algorithm performance (alternant response). (MP4)
