# Supplementary material for: A novel methodology to characterize beat-to-beat alternations in whole-cell calcium currents
Source: PLoS One. 2026 Jan 28;21(1):e0339890. doi: 10.1371/journal.pone.0339890 (PMC12851451; doi:10.1371/journal.pone.0339890)
Supplement: S1 File — (PDF) [file pone.0339890.s001.pdf]

Supplementary Material

A novel methodology to characterize beat-to-beat alternations in whole-cell calcium currents

Xavier Marimon[1,2*], Miguel Cerrolaza[3], Carmen Tarifa[4], Leif Hove-Madsen[5,6,7]

[1] Department of Strength of Materials and Structural Engineering, Universitat Politècnica de Catalunya (UPC BarcelonaTECH), Barcelona, Spain

[2] Institut de Recerca i Innovació en Salut (IRIS), Barcelona, Spain

[3] School of Engineering, Science and Technology, Universitat Internacional de Valencia (VIU), Valencia, Spain

[4] Spanish National Center for Cardiovascular Research (CNIC), Madrid, Spain

[5] Instituto de Investigaciones Biomédicas de Barcelona (IIBB-CSIC), Barcelona, Spain

[6] Biomedical Research Institute Sant Pau (IIB Sant Pau), Barcelona, Spain

[7] Centro de Investigación Biomédica en Red, Enfermedades Cardiovasculares (CIBERCV), Madrid, Spain

* Corresponding author
E-mail: xavier.marimon@upc.edu

S1. Preparation of human atrial cardiac tissue samples

Human atrial cardiomyocytes (Huma Atrial Myocyte, HAM) have been used in experimental tests. The human tissue sample must be extracted during cardiac revascularization (bypass) operations with extracorporeal circulation at the Cardiology and Cardiac Surgery Unit of the Hospital de la Santa Creu i Sant Pau (Barcelona, Spain). Prior to cannulation of the equipment, the tissue sample is removed from the atrium, stored in cold oxygenated Tyrode solution, and transported to the laboratory. Patients who received treatment with calcium antagonists have been excluded from the study. The protocol has been approved by Hospital de la Santa Creu i Sant Pau Ethics Committee and complies with the Helsinki Declaration of Ethical Principles of the World Medical Association (WMA). All samples were taken with the informed consent of the donors.

S1.1 Isolation of cardiomyocytes

Once the tissue sample has been extracted by cutting 1x1 mm fragments, cardiomyocytes are isolated by enzymatic digestion. In order to prevent the degradation of the atrium tissue, it is immersed in a solution without calcium, called calcium-free Tyrode solution (see Table S.1). Then, 30 mM monoxyma-2,3-butanedione, $C_4H_7NO_2$, is added immediately after extraction.

S1.2 Enzymatic Digestion

Tissue cuts are carried out in the calcium-free Tyrode solution (see Table S.1). The first enzymatic digestion is then carried out for a 30-min period at a temperature 35 ºC. After 30 minutes, the tissue is removed from the first enzyme solution to interrupt the first digestion and washed with a Tyrode solution of calcium with 5%

BSA, fatty acid-free. Once the tissue has been cleaned, the cells are disintegrated using a 3-ml Pasteur pipette. The remaining non-disintegrated tissues undergo successive enzymatic digestions lasting 15 min. The Tyrode solution is used for later digestions (see Table S.2).

Table S.1. Composition of the calcium-free Tyrode solution

Solution	Composition	pH	Temperature
Tyrode (without C_aCl_2)	88 mM NaCl<br>5.4 mM KCl<br>1.1 mM $MgCl_2$<br>0.3 mM NaH_2PO_4<br>4 mM $NaHCO_3$<br>10 mM HEPES<br>10 mM glucose<br>88 mM sucrose<br>5 mM Na^+ pyruvate<br>20 mM taurine	7.4 (with NaOH)	35 ºC

Table S.2. Composition of enzymatic digestion solutions

Solution	Composition	Time	Temperature
First digestion	Tyrode (without $CaCl_2$)<br>1.8 mg/ml collagenase<br>0.42 mg/ml protease<br>2 mg/ml BSA	30 min	35 ºC
Subsequent digestions	Tyrode (without $CaCl_2$)<br>0.6 mg/ml collagenase<br>2 mg/ml BSA	15 min	35 ºC

After all the tissue has been disintegrated, the obtained cardiomyocytes are stored in a maintenance solution (see Table S.3), and the calcium concentration in the solution is gradually increased to 1 mM. Cells obtained will no longer be used for experimentation for a time window of 10 hours.

Table S.3. Composition of the cardiomyocyte maintenance solution

Solution	Composition
Maintenance	Tyrode (without $CaCl_2$)<br>1 ml MEM vitamines<br>1 ml penicillin-streptomycin<br>2 ml MEM essential aminoacids (50X)<br>1 ml MEM essential aminoacids (100X)<br>500 mg BSA

S1.3 Selection criteria for cardiomyocytes

Elongated cardiomyocytes with striation and no granulation were selected. To prevent cardiomyocytes adhesion to the Petri dish, a pre-treatment is performed on the plates with Bovine Serum Albumin (BSA) lyophilised in the extracellular solution. To perform the experiment, the plaque solution is replaced by the extracellular solution without BSA, and then a few drops of the solution containing the suspended cardiomyocytes are added. The composition of the solutions of both the intracellular and extracellular medium of the cardiomyocyte is shown in Table S.4.

Table S.4. Composition of intracellular and extracellular medium

Medium	Composition	pH	Temperature
Intracellular	1 mM $MgCl_2$<br>47 mM CsCl<br>3 mM Mg_2ATP<br>109 mM aspartic acid<br>5 mM Na_2-phosphocreatine<br>0.42 mM Li_2GTP<br>10 mM HEPES<br>250 µg/ml amphotericin B	7.2<br>(with NaOH)	35 ^{o}C
Extracellular	2 mM $CaCl_2$<br>127 mM NaCl<br>1.8 mM $MgCl_2$<br>0.33 mM NaH_2PO_4<br>4 mM $NaHCO_3$<br>10 mM HEPES<br>10 mM glucose<br>5 mM pyruvic acid<br>5 mM TEA	7.4<br>(with KOH)	35 ^{o}C

S1.4 Patch-clamp recording

Calcium ion currents (*Ca^{2+}*) are recorded using the patch-clamp technique in the whole-cell configuration in freshly isolated myocytes using the HEKA amplifier model [1] EPC 10 USB. The selected cardiomyocyte is lifted from the Petri dish bottom and placed in front of a capillary system by perfusion.

Once the cardiomyocytes have properly settled, the micropipette is placed close to the selected cardiomyocytes, and a slight negative pressure is applied to catch it. When the initial resistance of the micropipette (*Rp*), 1-2.5 mΩ, reaches 1 GΩ this is indicative that a correct contact seal has been formed between the micropipette tip inner-surface and the cell membrane. After the seal is properly formed, the negative pressure is no longer applied since it is unnecessary. Both the bath and pipette solutions are shown in Table S.5.

Table S.5. Composition of patch-clamp recording solutions

Solution	Composition	pH	Temperature
Cell bath	2 mM $CaCl_2$<br>136 mM NaCl<br>1.6 mM $MgCl_2$<br>4 mM KCl<br>0.33 mM NaH_2PO_4<br>4 mM $NaHCO_3$<br>10 mM HEPES<br>5 mM glucose<br>5 mM pyruvic acid	7.4<br>(with NaOH)	35 ^oC
Pipette	1 mM $MgCl_2$<br>47 mM KCl<br>3 mM Mg_2ATP<br>109 mM aspartic acid<br>5 mM Na_2-phosphocreatine<br>0,42 mM Li_2GTP<br>10 mM HEPES<br>250 µg/ml amphotericin B	7.2<br>(with KOH)	35 ^oC

S2. Patch-clamp model

The full patch clamp electrical circuit used to simulate the cardiomyocyte biophysics and the patch clamp amplifier is shown in the Fig. S1. The electrical circuit has been implemented and simulated using the LTspice software [2]

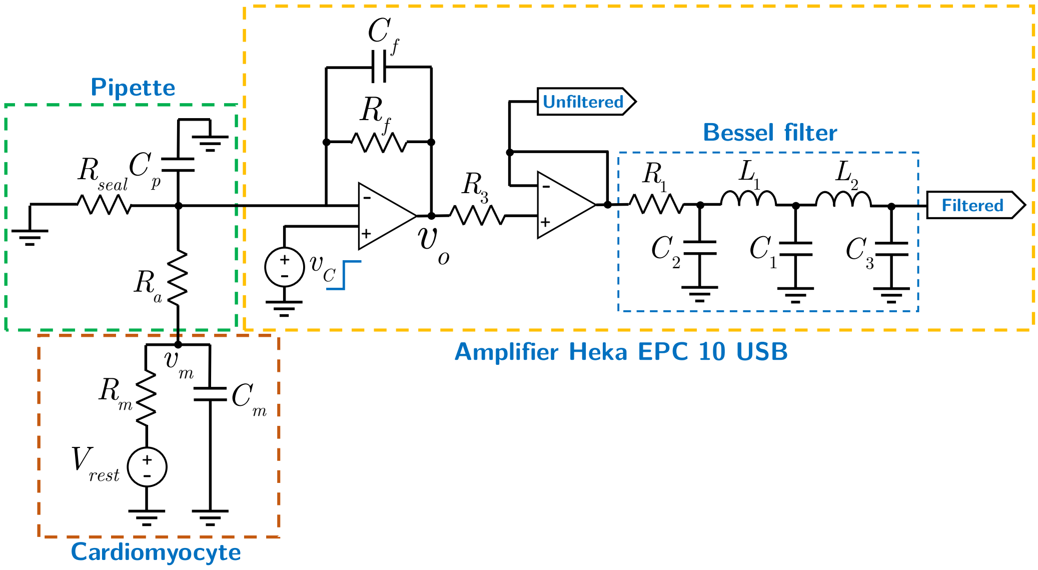

Fig. S1. Electrical circuit used for the numerical simulation of the patch-clamp experiment.

The electrical variables that characterize the cardiomyocyte used in the electrical model are:

- Membrane potential (v_m): potential difference in the cardiomyocyte membrane.
- Resting potential (V_{rest}): cardiomyocytes and cardiac Purkinje fibers [3] usually maintain a membrane potential close to the equilibrium potential of potassium (K^+) around -90 mV. The resting potential is simulated with a voltage source (see Fig. S2).
- Membrane capacitance (C_m): electrical capacity of the cell membrane and it describes how much charge is required to change the membrane voltage [4]. Larger cells with more membrane surface area have a larger capacity and require more charge to change their voltage. Several authors have measured a membrane capacity for individual cardiomyocytes between 100-400 pF in several species [5]. The membrane capacitance measured in the cardiomyocytes in our experiment is approximately 55 pF.
- Membrane resistance (R_m): resistance of the cell membrane [6-8]. It is determined by the properties of the ion-channels of the cell membrane. The membrane conductance G_m is defined as the inverse of the membrane resistance. The conductance increases proportionally according to the number of open ion-channels in the membrane.

The electrical elements that characterize the pipette are:

- Access resistance (R_a): resistance caused by the small open-entry tip of the micropipette. If the tip of the pipette is blocked, the resistance increases. This resistance is of order MΩ [9]. A voltage source that models the offset voltage introduced by the electrode-cell junction is often also added in series to R_a [10,11]. This access resistance is between 10 MΩ and 100 MΩ) [12].
- Seal resistance (R_{seal}): resistance formed by the seal between the cell's surface and the glass pipette. Ideally, high seal resistances in the gigaOhms (GΩ) range are sought.

- Pipette capacitance (C_p): capacitance of the glass wall of the micropipette that acts as a polarized capacitor [6].
- Pipette resistance (R_p): resistance between the amplifier and the micropipette's tip. This resistance is due to the resistance of the saline solution inside the micropipette [13]. It is commonly neglected because its low values.
- Series resistance (R_s): defined as the sum of all the pipette resistances, hence the sum of the non-biological resistances: R_a, R_p and R_{seal}. The access resistance, R_a, is the largest contributor to the series resistance. A high series resistance is bad for two reasons: it acts as a low-pass filter and it also acts as a voltage divider in series with the membrane resistance, R_m, resulting in a steady-state voltage error.

S3. Time-constants fitting

Time constants features, measure the rate at which the ion currents change over time. These features can provide insight into the kinetics of ion channel gating, and can be used to identify differences in the speed of ion channel opening and closing between cells or under different conditions. The inactivation phase of a channel occurs when the current of the studied channel disappears. Thus, the inactivation phase of calcium channels is the phase of disappearance of the L-type calcium current I_{Ca}. The parameter measured to characterize the inactivation rate is the time constant of the rise curve. This parameter was obtained by fitting the L-type calcium current curve to a simple exponential model of the type:

$$f(x) = a \cdot \left(1 - e^{-\frac{(x-b)}{\tau}}\right) + c \tag{S.1}$$

where a is the maximum transient current, c is the peak current, b is the time elapsed until the peak and τ is the time constant.

Figure S2 displays the fit of the L-type calcium current to a simple exponential model, from which the time constant, τ, can be obtained.

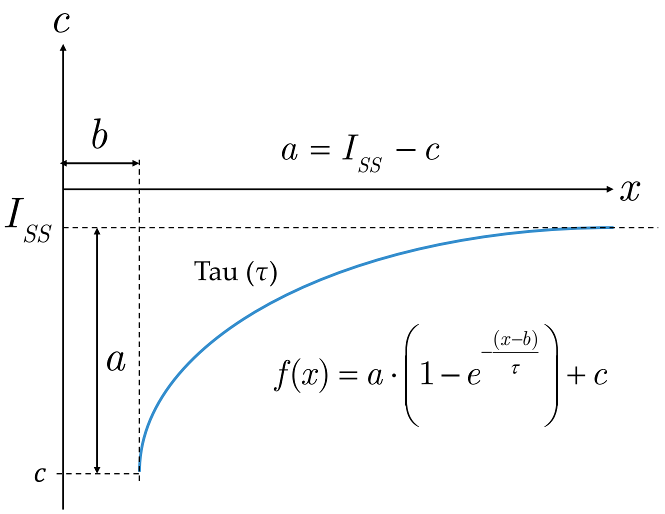

Fig. S2. Exponential model to fit the L-type calcium current, I_{ca}, used to determine the time constant τ.

The amplitude of the maximum transient current a is defined as:

$$a = I_{SS} - c \tag{S.2}$$

where I_{ss} is the steady state.

The case of tail current, I_{Tail}, is a bit more complex. Two types of adjustments have been evaluated. One based on a single exponential model and another model based on a double exponential. The single exponential model is similar to the case of the L-type calcium current, and is given by:

$$f(x) = a \cdot \left(1 - e^{-\frac{(x-b)}{\tau_1}}\right) + c \tag{S.3}$$

where τ_1 is the time constant.

In the case of the double exponential model, it is considered that the inactivation curve has two dynamics: a fast dynamic and a slow dynamic. Therefore, two-time constants are needed in this model. The fast time constant will be denoted as τ_1, while the slow time constant will be denoted as τ_2. Thus, the two time constants, τ_1 and τ_2, have been obtained by fitting the curve to a double exponential model of the type:

$$\begin{aligned} f(x) &= a_1 \cdot \left(1 - e^{-\frac{(x-b)}{\tau_1}}\right) + (1 - a_1) \cdot \left(1 - e^{-\frac{(x-b)}{\tau_2}}\right) + c \\ a_1 &= P \cdot 0.01 \cdot a \end{aligned} \tag{S.4}$$

where τ_1 is fast time constant, τ_2 is the slow time constant and P is the percentage of fast signal.

Figure S3 shows the parameters describing the tail current fit to a single and double exponential model.

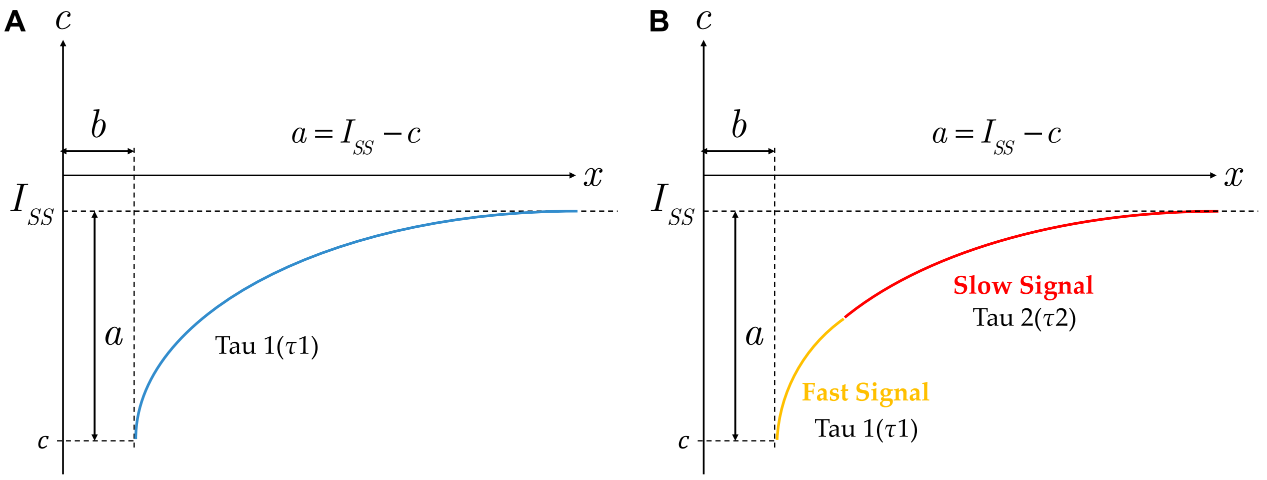

Fig. S3. Models to fit the tail current I_{Tail}. **A.** Single exponential model used to determine only one time constant τ_1. **B.** Double exponential model used to determine the two time constants: the fast time constant τ_1 and the slow time constant τ_2

S3.1. Time-constants fitting

Two types of adjustments have been evaluated. One based on a single exponential model and another model based on a double exponential. The time constants, τ values, are obtained by adjusting equations (S.3) and (S.4) for both single-exponential and double-exponential models. To assess the goodness of the Fit (GOF). The AIC criterion compares fit models from the perspective of entropy in information theory, as measured by Kullback-Leibler divergence [14-16]. For our case of normally distributed residuals, we can estimate the likelihood from the sum of squared estimate of errors (SSE), then the AIC becomes [16]:

$$AIC = 2 \cdot k + n \cdot \log\left(\frac{SSE}{n}\right) \tag{S.5}$$

where k is the number of estimated parameters, SSE is the squared estimate of errors, and n is the sample size of the model.

For the single exponential model, there are k=4 estimated parameters, while there are k=5 parameters for the double exponential model. To evaluate the accuracy of our models, we use a dataset of 24 patch-clamped cells (N=24) with a stimulus that has been repeatedly repeated successively 30 times ($Nsweep$=30). This dataset contains N_{Tail}=720 tail current values (single exponential model or double single exponential model).

S4. Model stability

In our model, the stability is primarily governed by an operational amplifier circuit with a parallel RC negative feedback network. To evaluate the range of the feedback resistor R_f and feedback capacitor C_f values where the op-amp circuit remains stable, we need to analyse the transfer function, and calculate the phase margin and gain margin, and determine the conditions for stability. Analytically, for the op-amp circuit with a parallel RC feedback network, the transfer function is given by:

$$H(s) = -\frac{R_f}{R_{in}} \cdot \frac{1}{1 + R_f \cdot C_f \cdot s} \tag{S.6}$$

where s is the Laplace variable representing the complex frequency $s=j\omega$.

The input resistance R_{in} is the total resistance measured by the amplifier, i.e. the sum of the diaphragm resistance R_{m} and the electrode resistance, described by the pipe resistance R_p and the access resistance R_a, using our measured values: $R_{\text{in}} = R_{\text{m}} + R_a + R_p$ =500 MΩ+15 MΩ+0 =515 MΩ. To ensure stability, we need to calculate the gain margin and the phase margin. To systematically evaluate the stability of our system, we perform a sweep over a range of R_f and C_f. Thus, for a given input resistance, R_{in} (fixed), we sweep over a range of values for the feedback resistor R_f and feedback capacitor C_f to determine the regions where the system remains stable (i.e. where the phase margin is above 45° and the gain margin is positive).

In this selection we use a feedback resistance of "Medium" gain range (500 MΩ) for the Heka amplifier, which is standard for whole-cell recordings, ensures that the system remains stable without introducing excessive amplification that could lead to instability or saturation in the signal processing. To check the stability for the case where the feedback capacitance C_f=0 and the feedback resistance R_f =500 MΩ, one can observe that the

transfer function simplifies because the feedback capacitance is zero. The transfer function becomes: $H(s)= R_f / R_{in}$, which represents a pure gain (no frequency dependence) because C_f=0 eliminates the dynamic (frequency-dependent) behavior.

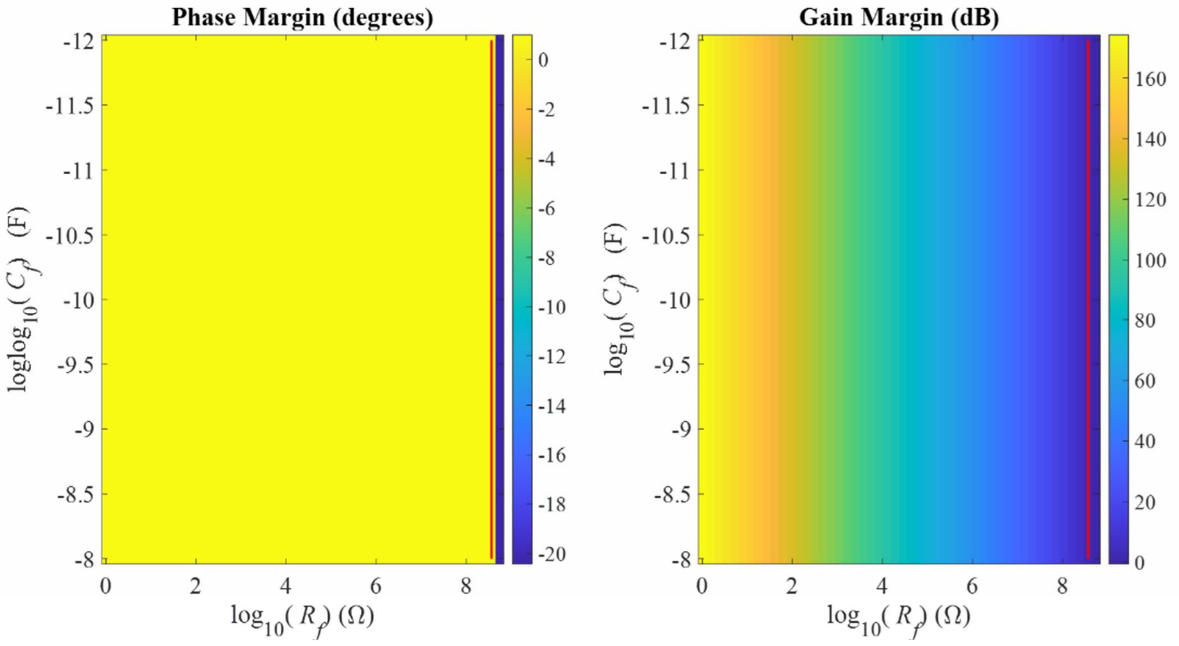

Fig. S4. Graphical representation of the stability regions by plotting the Phase Margin (left) and Gain Margin (right) on a logarithmic scale as a function of R_f and C_f. The input resistance Rin is defined as constant. The ranges of R_f and C_f are plotted on a logarithmic scale. The phase margin and the gain margin are displayed in decibels (dB).

This is why the denominator becomes 1 (no poles or frequency dependence). since the system has no frequency-dependent behavior, the phase margin might not give meaningful results. Since the system is purely resistive, there will be no poles or zeros introduced by the feedback network. This means that the gain margin should be infinite (or very large), as there is no frequency where the gain crosses 0 dB. Also, the phase margin may not be applicable (the phase might stay at 180° or -180°), since the system is not frequency-dependent. Therefore, the system is effectively stable because there are no dynamic components that could introduce oscillations. Moreover, the stability of the proposed electrical model is also ensured by the use of a fourth-order Bessel low-pass filter in the Heka amplifier, selected specifically to reduce oscillations and smooth the signal output. Bessel filters are known for their smooth phase response and lack of overshoot, making them effective for avoiding instability and excessive signal distortion. Moreover, the model accounts for noise by simulating realistic experimental conditions with additive Gaussian White Noise (GWN), which reflects real signal conditions without causing instability due to excessive noise or improper handling of stochastic fluctuations.

S5. Complementary results

The time constants do not seem to be the most significant data for calculating the alternation index CIdx. Only the index calculated with the time constant τ_1 shows statistically significant differences between cardiomyocytes with uniform and alternating response, with a p-value of $p \leq 0.05$ (*), as shown in Fig. S5.B. The rest of the time counter measurements used to calculate the alternation index C_{Idx} do not show statistically significant differences (n.s) between cardiomyocytes with a uniform or alternating response. (see Figs. S5.A-C).

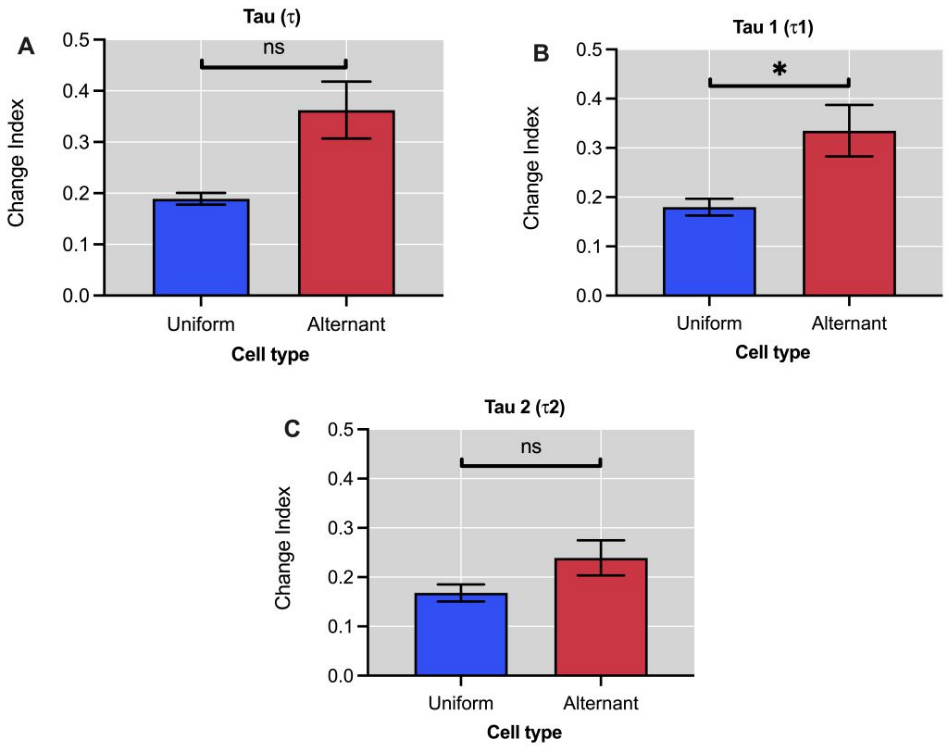

Fig. S5. The Alternation index CIdx for uniform and alternating type cardiomyocytes.
A. Alternation index calculated with the time constant of the calcium current τ. **B.** Alternation index calculated with the time constant of the fast tail current**. C.** Alternation index calculated with the time constant of the tail current

S6. Dataset description

A total of 10 distinct patient samples were analyzed in this study, from which N=24 single cardiomyocytes were recorded. Among these cells, 13 exhibited uniform electrical responses, while 11 displayed alternans. Because patient samples produced both alternant and uniform cells, the electrophysiological phenotype is not fixed at the patient level, and individual patients can transition between uniform and alternant behaviour over time. Table S.6 shows a summary of all recorded cardiomyocytes., showing the unique cell identifier (Cell ID), patient number, cell number, and electrical response phenotype (U = uniform; A = alternans).

Table S.6. Summary of all recorded cardiomyocytes.

Cell ID	Patient	Cell	Response
20110316_16_01	16	1	A
20110110_17_02	17	2	U
20110207_17_01	17	1	A
20110125_13_01	13	1	A
20110202_13_01	13	1	A
20110324_13_02	13	2	A
20110427_13_01	13	1	U
20110503_13_02	13	2	A
20110503_13_01	13	1	U
20110504_13_01	13	1	U
20110523_13_02	13	2	A
20110526_13_01	13	1	U
20110602_13_02	13	2	U
20130107_15_02	15	2	A
20101028_28_01	28	1	U
20130326_28_01	28	1	U
20140310_28_01	28	1	A
20150403_36_01	36	1	A
20111107_25_01	25	1	U
20110301_25_01	25	1	U
20180528_25_01	25	1	U
20180418_26_01	26	1	U
20140414_29_02	29	2	U
20140211_48_02	48	2	A

REFERENCES

1. Heka Elektronik GmbH 2024. https://www.heka.com. Ludwigshafen am Rhein, Germany
2. Analog Devices Inc. 2024. https://www.analog.com. Massachusetts,US.
3. Lopatin A, Nichols CG. Inward rectifiers in the heart: an update on I(K1). J Mol Cell Cardiol. 2001; 33(4):625-38. DOI: 10.1006/jmcc.2001.1344.
4. Neher E, Marty A. Discrete changes of cell membrane capacitance observed under conditions of enhanced secretion in bovine adrenal chromaffin cells. Proc. Nat. Acad. Sci. 1982; 79:6712-16. doi:10.1073/pnas. 79.21.6712.
5. Satoh H, Delbridge LM, Blatter LA, Bers DM. Surface:volume relationship in cardiac myocytes studied with confocal microscopy and membrane capacitance measurements: species-dependence and developmental effects. Biophys J. 1996; 70(3):1494-1504. doi:10.1016/S0006-3495(96)79711-4
6. Major G. Solutions for transients in arbitrarily branching cables: III. Voltage clamp problems. Biophys J. 1993; 65(1):469-91. doi: 10.1016/S0006-3495(93)81039-7.
7. Major G, Evans JD, Jack JJB. Solutions for transients in arbitrarily branching cables II. Voltage clamp theory. Biophys J. 1993a; 65(1):450-68. doi: 10.1016/S0006-3495(93)81038-5.

8. Major G, Evans JD, Jack JJ. Solutions for transients in arbitrarily branching cables: I. Voltage recording with a somatic shunt. Biophys J. 1993b; 65(1):423-49. doi:10.1016/S0006-3495(93) 81037-3.
9. Marty A, Neher E. Tight-Seal Whole-Cell Recording. In: Sakmann B & Neher E (Eds.) Single-Channel Recording. Springer. MA, pp. 31-52: 1995.
10. Neher E. Ion Channels for Communication Between and Within Cells. Science 1992; 256(5056):498-50. doi: 10.1126/science.1373906.
11. Neher E. Voltage Offsets in Patch-Clamp Experiments, in: E. Sakmann Bert and Neher (Ed.), Single-Channel Recording, Springer US, Boston, MA, pp. 147–153; 1995.
12. Major G, Evans JD. Solutions for transients in arbitrarily branching cables: IV. Nonuniform electrical parameters. Biophys J. 1994; 66(3):615–33. doi: 10.1016/S0006-3495(94)80836-7.
13. Hamill OP, Marty A, Neher E et al. (1981) Improved patch-clamp techniques for high-resolution current recording from cells and cell-free membrane patches. Pflugers Arch 391:85-100. doi.org/10.1007/BF00656997.
14. Akaike H. A new look at the statistical model identification. IEEE Trans on Autom. Control 1974; 19(6):716-23. doi: 10.1109/TAC.1974.1100705
15. Akaike H. Information Theory and an Extension of the Maximum Likelihood Principle. In: Parzen E et al (Eds) Selected Papers of Hirotugu Akaike. Springer. New York. pp. 199-213; 1998.
16. Burnham KP, Anderson DR. Model Selection and Multimodel Inference: A Practical Information- Theoretic Approach. 2nd ed. Springer, New York; 2002.
